# Mechanism of anti-tumor and anti-invasive function of SOCS isoform in colon cancer models

**DOI:** 10.1186/1878-5085-5-S1-A46

**Published:** 2014-02-11

**Authors:** Won Ki Kim, ji-Yoon Ryu, Byong Chul Yoo, Choong-Eun Lee

**Affiliations:** 1 Colorectal Cancer Branch, Division of Translational and Clinical Research I, Research Institute, National Cancer Center, Gyeonggi, 410-769, Republic of Korea; 2 Laboratory of Immunology, Department of Biological Science, Sungkyunkwan University, Suwon, 440-746, Republic of Korea

## 

Suppressor of cytokine signaling (SOCS) proteins are the negative regulators in cellular signal transduction system. SOCS family proteins consisting of 8 proteins (CIS and SOCS1-7) inhibit JAK/STAT protein activity to maintain proper cytokine functions. Initially identified for the negative feed-back function of cytokine signal transduction, suppressors of cytokine signaling (SOCS) have emerged as multi-functional protein involved in the regulation of development, apoptosis and oncogenesis. Recent reports showed that SOCS can be also affected by cytokine and other cell signaling factors to regulate cancer development and progression as well as cell death. The present study was aimed to investigate various effects of SOCS1 protein on the tumor suppressive function. We have found that SOCS1 promotes radiation-induced apoptosis of colon cancer cells through cell cycle arrest at G1 involving activation of p53 and p21 induction. SOCS1 also exhibits radiosensitization effects on the growth of implanted colon tumor cells in nude mice. As a mechanism of anti-tumor action of SOCS, we now present the data on the suppressive effects of SOCS1 on tumor cell migration and invasion. Notably SOCS1 over-expression and knock-out inversely regulates the expression of EMT markers such as E-cadherin and vimentin along with modest morphological change of cells from epithelial to fibroblast shape, representing EMT process. Data on the invasion and EMT signaling pathways suggest that SOCS1 potentially regulates tumor cell invasion and metastasis in vitro and in vivo in colon cancer models. SOCS1 may be not only a useful predictive factor for radiation response but also a possible drugable target for human colon cancer.

